# Noncontact Cardiac Activity Detection Based on Single-Channel ISM Band FMCW Radar

**DOI:** 10.3390/bios13110982

**Published:** 2023-11-12

**Authors:** Kui Qu, Lei Wei, Rongfu Zhang

**Affiliations:** 1 School of Physics and Electronic Engineering, Fuyang Normal University, Fuyang 236037, China; wei.lei.fy@foxmail.com; 2 School of Optical-Electrical and Computer Engineering, University of Shanghai for Science and Technology, Shanghai 200093, China; zrf@usst.edu.cn

**Keywords:** cardiac movement, respiratory harmonics, noncontact detection, radar

## Abstract

The heart is an important organ that maintains human life activities, and its movement reflects its health status. Utilizing electromagnetic waves as a sensing tool, radar sensors enable noncontact measurement of cardiac motion, offering advantages over conventional contact-based methods in terms of comfort, hygiene, and efficiency. In this study, the high-precision displacement detection algorithm of radar is applied to measure cardiac motion. Experimental is conducted using a single out-channel frequency modulated continuous wave (FMCW) radar operating in the ISM frequency band with a center frequency of 24 GHz and a bandwidth of 150 MHz. Since the detection signal is influenced by both respiratory and heartbeat movements, it is necessary to eliminate the respiratory signal from the measurement signal. Firstly, the harmonic composition of the respiratory signal is analyzed, and a method is proposed to calculate the parameters of the respiratory waveform by comparing the respiratory waveform coverage area with the area of the circumscribed rectangle. This allows for determining the number of respiratory harmonics, assisting in determining whether respiratory harmonics overlap with the frequency range of the heartbeat signal. Subsequently, a more accurate cardiac motion waveform is extracted. A reference basis is provided for extracting cardiac health information from radar measurement waveforms by analyzing the corresponding relationship between certain extreme points of the waveform and characteristic positions of the electrocardiogram (ECG) signal. This is achieved by eliminating the fundamental frequency component of the heartbeat waveform to emphasize other spectral components present in the heartbeat signal and comparing the heartbeat waveform, the heartbeat waveform with the fundamental frequency removed, and the heartbeat velocity waveform with synchronized ECG signals.

## 1. Introduction

The heart maintains normal vital activities by pumping blood to the whole body through contraction and relaxation. The health conditions of the heart itself and certain other organs can cause the heart to exhibit different motion states. While doctors commonly employ auscultation as an initial diagnostic method for assessing heart health, it fails to provide precise information about cardiac motion. Traditional methods of accurately measuring cardiac activity signals primarily involve the use of contact electrodes to measure ECG signals. However, these measurement instruments are complex to operate and require specialized skills, and they have limitations in terms of hygiene and flexibility. Cardiac motion produces macroscopic and measurable periodic fluctuations in the chest. Radar can realize noncontact measurement of cardiac activity states with its noncontact ability for high-precision displacement measurement. The radar frequencies commonly used to detect vital signs are mainly concentrated between 2 GHz and 100 GHz [1,2,3,4,5,6,7,8], with wavelengths ranging from 3 mm to 150 mm. Electromagnetic waves in this frequency range have good penetrability, allowing them to easily pass through ordinary media such as clothing and bedding. The shorter wavelengths provide microwave radar with a highly sensitive perception capability for detecting subtle movements, which endows microwave radar with unique advantages in noncontact monitoring of vital signs.

For FMCW radar, the phase of the beat signal from the target varies linearly with distance. When the target’s position changes by half a wavelength relative to the radar, the phase will shift by 2π. Phase measurement offers high sensitivity to motion. The methods for solving phase include small-angle approximation [9], orthogonal demodulation [10], and arctangent demodulation [11]. The arctangent demodulation method is widely used due to its highest accuracy in motion reconstruction, although it requires the elimination of direct current (DC) offset. In addition, since the phase is obtained through the inverse operations of trigonometric functions, which have periodicity, it is necessary to unwrap the calculated phase. There are two main methods for phase unwrapping. The first one is the traditional method, which determines whether the phase difference between two consecutive sampling points is greater than π or less than −π in order to decide whether the current phase should be incremented or decremented by 2π. This judgment process needs to be applied throughout the entire phase sampling procedure. The second method is known as the differentiate and cross multiply (DACM) algorithm [12,13,14], which calculates the discrete accumulation expression of the phase by first differentiating and then integrating it. Compared to the traditional algorithm, this approach does not require phase difference judgment, resulting in higher execution efficiency. The arctangent demodulation method requires two orthogonal signals. For FMCW radar, the in-phase/quadrature-phase (I/Q) dual output channel signals on the hardware can be used to form a complex beat signal, and then the Fourier transform is done on the complex beat signal and the real and imaginary parts of the obtained result are used as two orthogonal signals. Alternatively, the Fourier transform of the signal from a single out-channel can be used to obtain the real and imaginary parts for constructing the orthogonal signals. The single out-channel structure is simpler, aiding in device miniaturization while also offering the advantages of low cost and low power consumption. In this paper, the accurate calculation method for displacement under the single out-channel condition as described in [15] was employed.

Under normal breathing conditions, the amplitude of the chest surface vibrations caused by respiration is much greater than that of the heartbeat. Therefore, the measured vibration signal mainly reflects the fluctuation characteristics of the respiratory. Most literature simplifies the heartbeat and respiratory motions as harmonic motions in different frequency ranges [16,17,18,19,20,21]. If this assumption is true, the two motions can be separated in the frequency domain as long as the spectral resolution is sufficient. However, the respiratory and heartbeat motion signals not only overlap in the time domain but also the high-frequency harmonics of the respiratory signal may enter the frequency range of the heartbeat signal so that the traditional low-pass and high-pass filtering methods produce errors when separating respiratory and heartbeat signals. In [22], the respiratory waveform was determined through empirical formulas, and then it was removed from the measured signal to obtain the heartbeat signal and has achieved good results. Most studies on radar-based heartbeat signal measurement primarily focus on the average heart rate during the observation period [23,24,25,26,27,28], resulting in insufficient exploration of cardiac health information. Thus, it is necessary to conduct more in-depth research on the heartbeat signal to further uncover valuable insights into cardiac health.

In this study, a single in-phase out-channel FMCW radar operating in the ISM band with a central frequency of 24 GHz and a bandwidth of 150 MHz was used for the detection of cardiac activity signals. The ISM band is a set of frequency bands designated by the International Communication Union for use by industrial, scientific, and medical institutions without requiring a license or fees, making it more practical. The adoption of a single-channel configuration simplifies the hardware setup, enhancing system stability. In order to eliminate the harmonic components of respiratory signals, an algorithm has been proposed that determines the harmonic constituents of these signals by calculating the ratio of the area covered by the respiratory waveform to the circumscribed rectangle. This method is easy to implement and shows good precision in practice. Reference [22] extracted further cardiac health information by marking feature points on the heartbeat waveform while performing differential operations on the waveform to locate these feature points can be effective; this approach’s drawback is its high sensitivity to noise, which can adversely impact the result stability. In the pursuit of more stable feature points, the fact that the heartbeat waveform is not a simple harmonic wave of a single frequency is notable. While the amplitude of the fundamental frequency is the highest, other harmonic components—with smaller amplitudes—also contain relevant cardiac health information. This study proposes the removal of fundamental waves from the heartbeat waveform to highlight the harmonic components that may be masked. By comparing the obtained waveform with the synchronous ECG signal, the corresponding relationship between certain feature points in the waveform and characteristic locations in the ECG signal was analyzed, which provides a valuable approach to extract more information from radar cardiac motion signals.

## 2. Measurement Method

### 2.1. Single Out-Channel FMCW Radar Displacement Measurement Method

**Figure 1:**
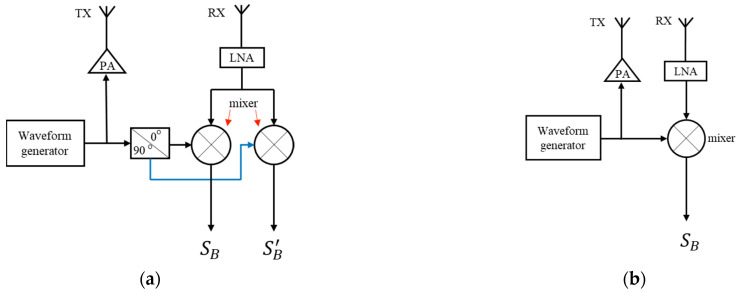
The schematic diagram of an FMCW radar. (**a**) Dual output channels; (**b**) Single output channel.

The waveform generator of the FMCW radar generates the chirp signal, which is then amplified by the power amplifier (PA) and emitted through the transmitting antenna (TX). The receiving antenna (RX) captures the echo signal, which is subsequently filtered and amplified by the low-noise amplifier (LNA). The mixing of the filtered echo signal and the transmitted signal yields the in-phase beat signal $S_{B}$. Similarly, the mixing of the echo signal with the 90° phase-shifted transmitted signal results in the quadrature-phase beat signal $S_{B}^{\prime }$. The expressions for the two types of beat signals are as follows.

(1) $$S_{B}=A\;\cos(\frac{4\pi \gamma R_{d}}{c}t+\frac{4\pi f_{0}R_{d}}{c}),$$

(2) $$S_{B}^{\prime }=A\;sin(\frac{4\pi \gamma R_{d}}{c}t+\frac{4\pi f_{0}R_{d}}{c}).$$

where A is the amplitude, $f_{0}$ is the start frequency, $R_{d}$ is the distance between the target and the antenna, c is the propagation velocity of electromagnetic wave, and γ is the frequency slope, $\gamma =B_{w}/T$, where $B_{w}$ is the bandwidth, and T is the duration of a chirp. The traditional FMCW radar with the I/Q dual output channel on the hardware can be used to form a complex beat signal. However, when the hardware is simplified to include only one output channel, there is only one beat signal output. The schematic diagrams for the two output configurations are illustrated in Figure 1.

Figure 1a shows the traditional dual output channel structured radar system. Figure 1b illustrates the single out-channel radar system. The single out-channel reduces one signal path, resulting in a simpler hardware structure. With the aid of specialized signal processing techniques, comparable accuracy in displacement measurement can still be achieved [15].

For single out-channel, applying the *N*-point DFT to the beat signal $S_{B}$ and consider the positive half side of the spectra only, we have

(3) $$F_{N}\left(R_{d},k\right)=\frac{1}{N}\sum_{n=0}^{N-1}S_{B}\cdot \exp(-j(2\pi f_{k}t_{n})),$$

where $t_{n}=nT/N$ is discrete sampling time, *n* is the number of sampling points, N is the total number of samples in a chirp. The discrete frequency is $f_{k}=k/T$, where *k* is the discrete frequency number, 1≤k≤N, k∈Z. For $F_{N}\left(R_{d},k\right)$, taking its value when N tends to infinity is a good approximation, as shown in Equation (4).

(4) $$F\left(R_{d},k\right)=\underset{N\to \infty }{\lim}F_{N}\left(R_{d},k\right),$$

Substitute (1) into Equation (4), according to [15], the real part and image part of $F\left(R_{d},k\right)$ can be obtained as

(5) $$F_{\;Im}\left(R_{d},k\right)=\frac{2B_{w}Rc}{4\pi {B_{w}}^{2}{R_{d}}^{2}-k^{2}\pi c}\sin\left(\frac{2\pi B_{w}R_{d}}{c}\right)\cdot \cos(\frac{4\pi R_{d}}{\lambda_{c}}),$$

(6) $$F_{Re}\left(R_{d},k\right)=\frac{kc^{2}}{4\pi {B_{w}}^{2}R^{2}-k^{2}\pi c}\sin\left(\frac{2\pi B_{w}R_{d}}{c}\right)\cdot sin(\frac{4\pi R_{d}}{\lambda_{c}}),$$

where $\lambda_{c}$ is the center wavelength of the transmitted wave, $\lambda_{c}={(f}_{0}+B_{w}/2)/c$. The value of N should be large enough to ensure that $F_{N}\left(R_{d},k\right)$ converges to the required accuracy. The beat signal of stationary objects will be processed into a complex DC offset based on (3), denoted as $A_{0}+{jB}_{0}$. In reference [15], it was demonstrated that when the displacement of the target satisfies $\left|\Delta R_{d}\right|\leq \lambda_{c}/2$, Equations (5) and (6) can be written as

(7) $$F_{Im}\left(R_{d},k\right)=A_{0}+A_{k}\cdot \cos\frac{4\pi R_{d}}{\lambda_{c}},$$

(8) $$F_{Re}\left(R_{d},k\right)=B_{0}+B_{k}\cdot \sin\frac{4\pi R_{d}}{\lambda_{c}},$$

Obviously, the trajectories of $F_{Im}\left(R_{d},k\right)$ and $F_{Re}\left(R_{d},k\right)$ on the complex plane are approximated as ellipses. By employing a trajectory fitting algorithm, the parameters of the ellipse can be obtained, followed by the elimination of the DC offset. Subsequently, the phase variation of the moving target can be obtained through the arctangent demodulation and unwrapping algorithm. By leveraging the relationship between phase variation and displacement,

(9) $$\Delta \varphi =\frac{4\pi \Delta R_{d}}{\lambda_{c}},$$

The target’s displacement ${\Delta R}_{d}$ can be determined by

(10) $$\Delta R_{d}=\frac{\lambda_{c}\Delta \varphi }{4\pi }.$$

### 2.2. Harmonic Analysis of Respiratory Signals

#### 2.2.1. Empirical Formulas for Breathing Signals

The parameters related to normal breathing and heartbeat [29,30,31] are shown in Table 1.

According to Table 1, the fundamental frequencies of the two movements are in different frequency ranges. The fundamental frequency of the respiratory signal is relatively low and can be extracted by applying a low-pass filter. However, the higher harmonics of the respiratory signal may enter the frequency range of the heartbeat signal. In view of the characteristics of the respiratory waveform, an empirical formula was proposed in [32]. In order to facilitate harmonic analysis, this formula was further modified in [22], and the revised formula is as follows:

(11) $$y_{\mathrm{bre}}=\cos^{2N_{\mathrm{bre}}}\left(\pi f_{bre}t\right),$$

where $f_{bre}$ is the fundamental frequency of the respiratory signal, $N_{bre}$ is a positive integer. In [22], the positive direction is defined as the direction toward the radar. However, in this paper, the direction away from the radar is considered as the positive direction. When the target moves in the direction away from the radar, the distance relative to the radar increases, and it is more in line with the habit of understanding to choose this direction as the positive direction. Under this convention, the chest displacement during exhalation is positive, corresponding to a positive slope in the vibration waveform. Conversely, during inhalation, the slope of the vibration waveform is negative. During normal respiration, there is a slightly longer period of smooth transition from exhalation to inhalation, which should appear above the vibration waveform. To meet the convention of positive direction, Equation (11) has been modified as follows:

(12) $${y_{bre}=A}_{bre}\left(1-\cos^{2N_{\mathrm{bre}}}\left(\pi f_{bre}t\right)\right),$$

where $A_{bre}$ is the amplitude. Using Euler’s formula, Equation (12) can be written as

(13) $${y_{bre}=A}_{bre}-A_{bre}{\left(\frac{e^{-j\pi f_{bre}t}-e^{j\pi f_{bre}t}}{2}\right)}^{2N_{bre}},$$

According to the binomial theorem, Equation (13) can be written as

(14) $${y_{bre}=A}_{bre}(1-\frac{C_{{2N}_{bre}}^{N_{bre}}}{4^{N_{bre}}})-\frac{2A_{bre}}{4^{N_{bre}}}\sum_{i=0}^{N_{bre}-1}C_{{2N}_{bre}}^{i}\cos\left(2\pi f_{bre}(N_{bre}-i)t\right).$$

It can be seen that the function $y_{bre}$ is composed of a DC component and $N_{bre}$ single-tone functions. When the index i is $N_{bre}-1$, the corresponding function frequency is the fundamental frequency $f_{bre}$; The remaining $N_{bre}-1$ single-tone functions represent the harmonics of the respiratory movement, with the highest harmonic frequency being $N_{bre}f_{bre}$.

**Figure 2:**
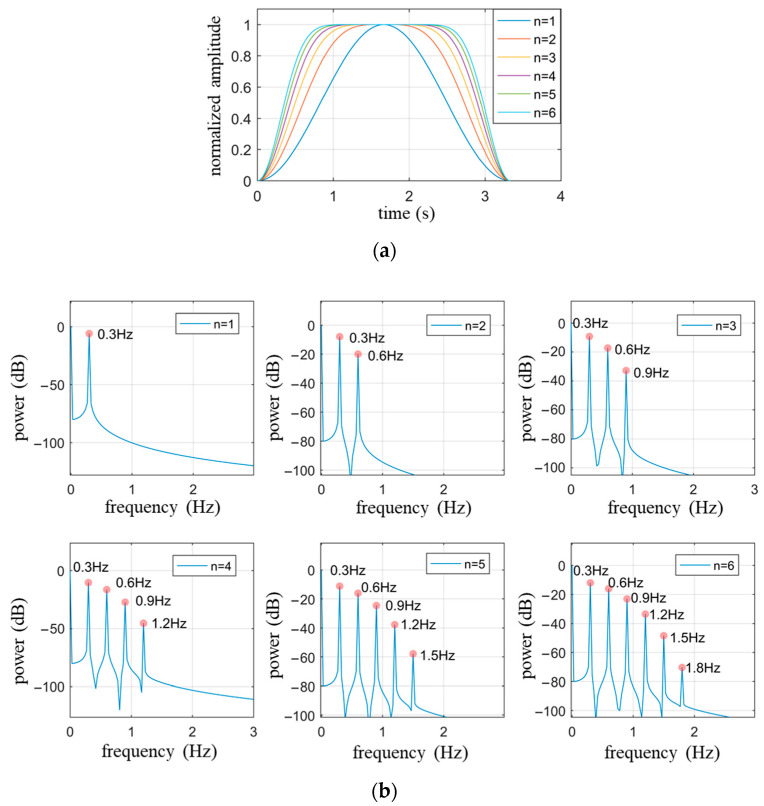
The waveform and spectrum of Equation (12) when $A_{bre}=1$, $f_{bre}=0.3\;Hz$, $N_{bre}\leq 6$. (**a**) Waveform within a respiratory cycle time; (**b**) The power spectrum of respiratory waveforms with different values of $N_{bre}$ corresponding to (**a**).

Figure 2a illustrates the respiratory waveform within one respiratory cycle described by Equation (12) as $N_{bre}$ gradually changes from 1 to 6, with an amplitude set to 1 and a fundamental frequency ($f_{bre}$) of 0.3 Hz. It is observed from Figure 2a that the value of $N_{bre}$ determines the proportion of time occupied by the smooth transition period within one respiratory cycle, where a larger $N_{bre}$ corresponds to a longer smooth transition period. Figure 2b presents the power spectrum of each respiratory waveform in Figure 2a, demonstrating that the number of harmonics is $N_{bre}-1$, consistent with theoretical analysis.

When extracting the heartbeat signal using a high-pass filter, it is essential to consider whether there are respiratory harmonics exceeding the cutoff frequency of the high-pass filter. Taking the cutoff frequency of 0.8 Hz as an example, Figure 2 indicates that when $N_{bre}\geq 3$, the harmonics of the respiratory signal are included in the heartbeat signal, thereby affecting the restoration of the heartbeat signal.

#### 2.2.2. Determination of the Number of Harmonics in Respiratory Waveforms

**Figure 3:**
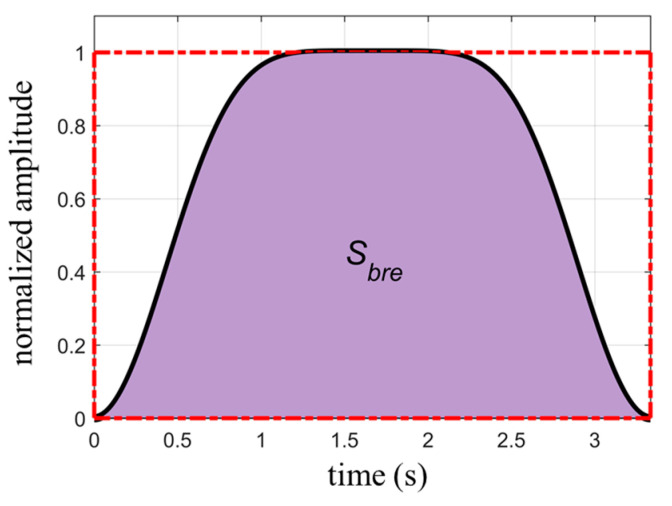
The area covered by one respiratory cycle (dark area) and the circumscribed rectangle of the waveform (red dashed box).

To obtain the value of $N_{bre}$ for the respiratory waveform, examining the area covered by one respiratory cycle described by Equation (12) yields the following expression:

(15) $$S_{bre}=\int_{0}^{1/f_{bre}}y_{bre}(t)dt=(1-\frac{C_{{2N}_{bre}}^{N_{bre}}}{4^{N_{bre}}})A_{bre}/f_{bre},$$

where $S_{bre}$ is the area covered by one respiratory cycle. The duration of a respiratory cycle is $1/f_{bre}$, and the circumscribed rectangle area of the waveform is ${S_{rec}=A}_{bre}/f_{bre}$. Figure 3 shows a schematic diagram of the two types of areas. The ratio between $S_{bre}$ and $S_{rec}$ is given by:

(16) $${r=S_{bre}/S}_{rec}=1-\frac{C_{{2N}_{bre}}^{N_{bre}}}{4^{N_{bre}}},$$

The amplitude of the waveform and the number of respiratory cycles contained in the calculation are common factors of $S_{bre}$ and $S_{rec}$, which cancel out in the ratio presented in Equation (16). Therefore, under stable respiration, the ratio r is independent of the waveform amplitude and the number of respiratory cycles. It is solely dependent on the value of $N_{bre}$. Table 2 provides the values of r corresponding to the first ten values of $N_{bre}$.

While individuals can briefly control their respiration, they cannot regulate their heartbeat. Therefore, the measured respiratory waveform will inevitably be superimposed with heartbeat signals. Compared to the respiratory signal, the heartbeat signals have higher frequencies and smaller amplitudes, resulting in regular fluctuations superimposed on the respiratory waveform. The variations in waveform area caused by the “rise” and “fall” of the heartbeat cancel each other out. Thus, the area calculated directly from the measured waveform is basically the same as the area of the actual respiratory waveform. When determining the area of the waveform’s circumscribed rectangle, the upper of the rectangle can be set as the average of the vertical coordinates of the fluctuations in the upper part of the waveform, while the bottom can be defined as the average of the vertical coordinates of all the valley points in the waveform. The length of the rectangle corresponds to the duration of the captured waveform.

### 2.3. Estimation of Heartbeat Signals

There are two common methods for measuring heartbeat signals using radar. The first method requires the subject to hold their breath during the measurement, eliminating the interference caused by respiratory motion and obtaining a direct heartbeat waveform. This method yields the best measurement results but deviates from normal physiological activity, causing discomfort to the subjects and limiting the duration of measurement due to the inability to sustain breath-holding. The second method allows the subjects to breathe naturally during measurement. However, the waveform obtained using this method is a combination of respiratory and cardiac motion. Since the first method eliminates the interference from respiratory motion, the measured heartbeat waveform can serve as a reference for the heartbeat waveform obtained under the second method.

By performing harmonic analysis on the respiratory signal and applying a high-pass filter to remove the main components of the respiratory signal, the cutoff frequency of the high-pass filter can be set slightly below the minimum value of the cardiac fundamental frequency mentioned in Table 1, for example, 0.8 Hz. If there are respiratory harmonics with frequencies higher than the cutoff frequency, it is necessary to eliminate the respiratory harmonics that enter the passband of the filter.

Using a notch filter is the conventional method to eliminate narrowband waves. Although a notch filter has a good amplitude-frequency response, it can introduce phase distortions to other components in the spectrum, resulting in distorted details in the extracted heartbeat waveform. To achieve the goal of removing respiratory harmonics without distorting other waveform components, a frequency domain filtering method [33,34,35] is employed. Fourier transform converts a time domain signal to the frequency domain, where the frequency components and their corresponding amplitudes and phases can be obtained. By setting the amplitude of a certain frequency component to zero, the resulting signal waveform after Fourier inverse transform will no longer contain that frequency component, achieving the purpose of frequency domain filtering. To improve computational efficiency, this approach can be used concurrently with high-pass filtering. The specific process involves setting the spectral intensity below the cutoff frequency of the high-pass filter in the frequency spectrum of the measured signal to zero, achieving the goal of high-pass filtering. Then, the spectral intensity at the frequencies of the respiratory harmonics is set to zero, achieving harmonic elimination. Finally, an inverse Fourier transform is applied to obtain the time domain waveform of the signal. Compared to using a notch filter, this filtering method can accurately eliminate respiratory harmonic signals without affecting other frequency components in the passband. The trade-off is the additional computational step of inverse Fourier transformation.

## 3. Simulation

To verify the effectiveness of the proposed algorithm, the estimate of heartbeat waveforms under normal respiratory conditions is simulated.

**Figure 4:**
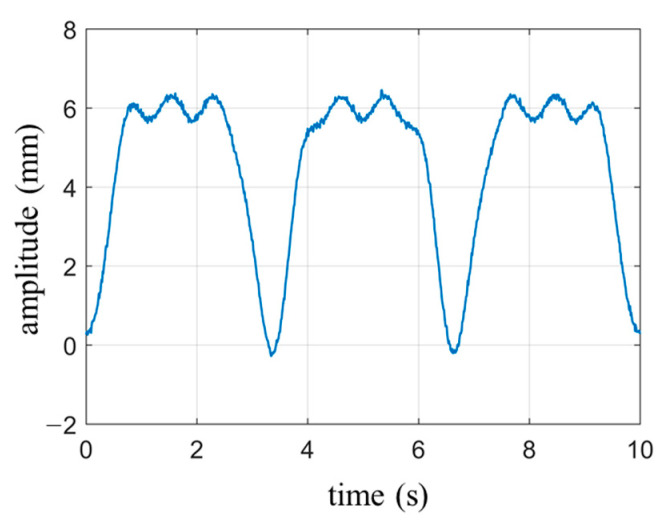
Simulate cardiopulmonary movement signals under normal breathing conditions.

The waveform of the respiratory signal is generated based on Equation (9), with the following parameter settings: $N_{bre}=3$, $A_{bre}=6\;\mathrm{mm}$, and $f_{bre}=0.3\;Hz$. The heartbeat signal is simulated using a single-tone wave with the parameters amplitude $A_{h}=0.3\;\mathrm{mm}$, frequency $f_{h}=1.3\;\mathrm{Hz}$ and the initial phase is zero. Gaussian white noise with a signal-to-noise ratio of 40 dB is added to the signal. The duration of the waveform is set to 10 s. The simulated waveform is shown in Figure 4. Since the amplitude intensity of the respiratory signal is 20 times stronger than that of the heartbeat signal, the overall waveform in Figure 4 primarily represents the periodic characteristics of the respiratory signal, with the heartbeat waveform being clearly visible only in the smooth region of respiration. The cutoff frequency for the high-pass filter is set to 0.8 Hz.

**Figure 5:**
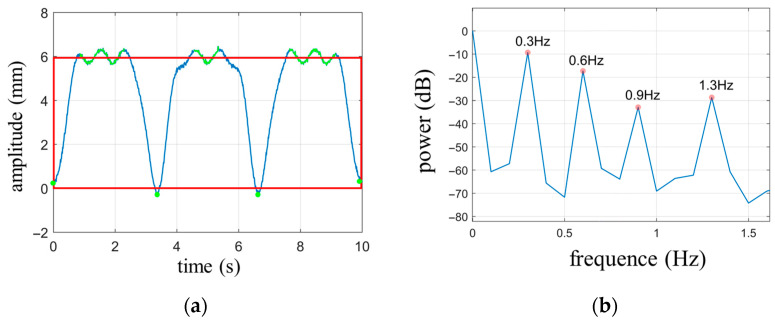
(**a**) The circumscribed rectangle of the respiratory waveform; (**b**) Power spectrum of simulated signals.

According to the calculation rules for the circumscribed rectangle area of the respiratory signal, the average of the vertical coordinates of the upper curve (indicated by the green portion in Figure 5a) yields the top coordinate of the rectangle (${rect}_{tp}$) as 5.96 mm. The average of all the valley points (marked with green dots at the bottom of the waveform in Figure 5a) determines the bottom coordinate of the rectangle (${rect}_{bm}$), which is 0.03 mm. The circumscribed rectangle is represented by a red outline. The area of the rectangle can be calculated as $S_{rec}=({rect}_{tp}-{rect}_{bm})\times 10=59.3$, while the covered area by the cardiopulmonary waveform is calculated as $S_{bre}=\sum \left(y(t_{i}){-rect}_{bm}\right)\times \Delta t=40.981$. In the digital signal, the waveform is discrete, so the area is obtained by summation, where y represents the wave function of cardiopulmonary movement and Δ*t* represents the discrete time interval. The ratio between the two areas is r=0.691, and according to Table 2, this corresponds to $N_{bre}=3$, which matches the set value.

Based on the power spectrum shown in Figure 5b, it can be observed that the minimum frequency where peaks are located, excluding the DC component, is 0.3 Hz, which corresponds to the fundamental frequency of respiration. Since $N_{bre}=3$, it is evident that the respiratory signal contains two harmonic components with frequencies of $\left(N_{bre}-1\right)f_{bre}=0.6$ Hz and $N_{bre}f_{bre}=0.9$ Hz. However, the frequency of the second harmonic component, 0.9 Hz, exceeds the cutoff frequency of the high-pass filter. Therefore, the signal obtained through high-pass filtering is a combination of the heartbeat signal and the second harmonic component of respiration.

**Figure 6:**
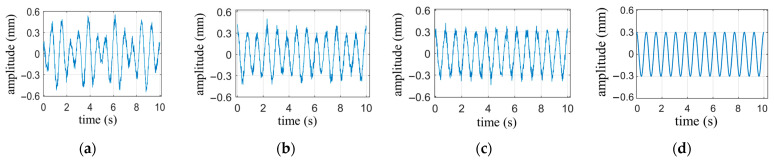
Comparison of heartbeat waveforms obtained by different methods. (**a**) heartbeat waveform obtained by high-pass filter; (**b**) Heartbeat waveform obtained by high-pass filter and notch filters; (**c**) Heartbeat waveforms obtained by frequency domain filtering method; (**d**) The simulated heartbeat waveform.

The result of filtering with a finite impulse response high-pass filter, which has a cutoff frequency of 0.8 Hz, a transition band of 0.1 Hz, and a stopband attenuation of 60 dB, is shown in Figure 6a. Compared with the simulated heartbeat waveform in Figure 6d, it can be seen that the high-pass filtered signal has significant distortion. Figure 6b illustrates the result after applying a notch filter to remove the 0.9 Hz respiratory harmonic from the signal filtered by the high-pass filter. It is evident that there are noticeable differences in peak values compared to the simulated waveform. Figure 6c illustrates the result using the frequency domain filtering method, which exhibits better peak stability compared to Figure 6b. The correlation coefficients between the three methods for obtaining the heartbeat waveform and the simulated waveform are 0.79, 0.91, and 0.98, respectively. Thus, the frequency domain filtering method demonstrates more precise filtering effectiveness. The notch filter used in the computation is a 16th-order Chebyshev Type II infinite impulse response filter with a −3 dB notch bandwidth of 0.01 Hz and a stopband attenuation of 80 dB.

## 4. Experimental Measurements

### 4.1. Experimental Setup

**Figure 7:**
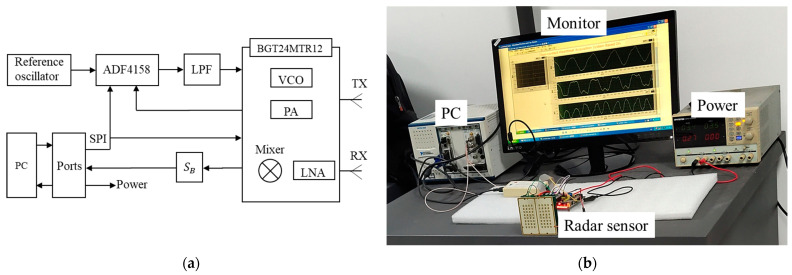
The radar structure and measurement setup: (**a**) The main components of the radar; (**b**) Measurement setup.

We constructed a 24-GHz FMCW radar system for motion detection. The waveform generator [36,37,38,39,40] of the radar is mainly composed of ADI’s ADF4158 chip (Norwood, MA, USA) and Infineon’s BGT24MTR chip (Munich, Germany), which are set up via SPI bus. The FMCW signal produced by the waveform generator is amplified by PA and transmitted to the object to be measured. The echo is amplified by the LNA and mixed with a sample of the transmitted signal to generate the beat signal, which is then converted into a digital signal through an ADC card. The radar adopts patch antennas, and the substrate used is Rogers 4350B. Figure 7a shows the main components of the radar, and Figure 7b shows the radar measurement setup.

**Figure 8:**
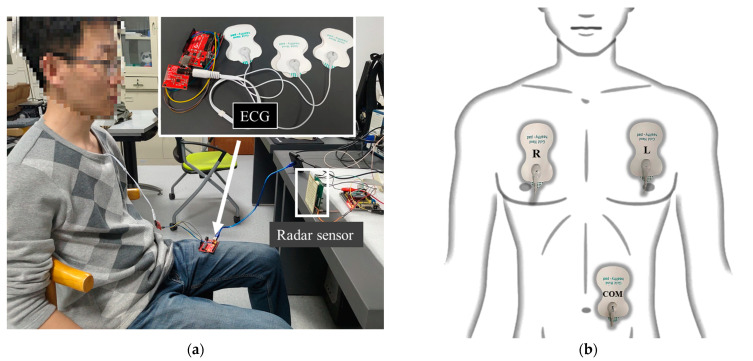
(**a**) Experimental setup and measurement environment; (**b**) The location of the three different electrodes of the ECG on the body. The letter “R” on the electrode represents “Right”, the letter “L” represents “Left”, and the letters “COM” represent “Communication”.

Simultaneously with radar measurements, the ECG signals of the subjects were obtained using the ECG monitoring kit manufactured by Keyes-Robot (Shenzhen, China). The kit consists of three electrode patches, which were applied to three different positions on the chest and abdomen during measurement, as shown in Figure 8b. The faint electrical signals captured by the electrodes were filtered and amplified by the ECG monitoring chip AD8232 from Analog Devices Inc. (Norwood, MA, USA). The analog signals were then digitized by the built-in analog-to-digital converter, and the data was uploaded to a PC through a USB interface. LabVIEW software (Version: 2021) is used to control the synchronous reception of radar signals and ECG signals, and the signal processing is performed by MATLAB (Version: 2021a). The experimental setup and measurement environment are illustrated in Figure 8a.

The motion signals obtained by radar and the electrical signals measured by the ECG recorder reflect changes in cardiac activity from two different perspectives. Since both signals originate from the same source, they should exhibit some level of consistency. The ECG signal is a well-established measurement technique, and by comparing the radar signals with the characteristic points of the ECG signal, it becomes possible to explore additional information related to cardiac health within the radar measurements.

### 4.2. Cardiac Activity Detection

The ventricle plays a primary role in pumping blood, and its cyclic changes in volume during contraction and relaxation generate mechanical vibrations that propagate to the surface of the body [41,42,43,44]. Consequently, the heartbeat signals detected by radar mainly reflect the motion state of the ventricle.

#### 4.2.1. The Heartbeat Waveform under Breath-Holding Condition

The subject sits quietly in a chair, with the radar positioned directly toward the subject’s chest. The measurements were conducted while the subject held his breath. The measurement duration was 10 s, with the radar positioned 0.4 m away from the chest. Simultaneously, ECG signals were also collected.

**Figure 9:**
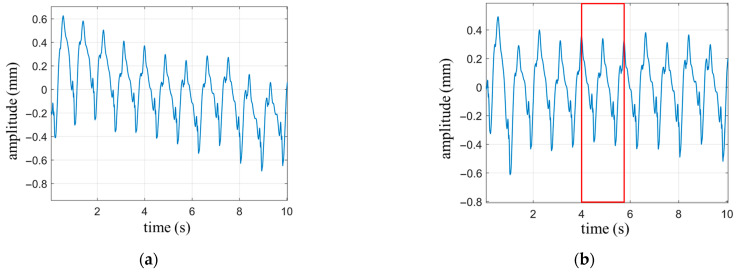
The heartbeat waveform under breath-holding condition. (**a**) Heartbeat waveform with drift; (**b**) Heartbeat waveform with drift removed through high-pass filtering.

Breathing is an instinctive response in humans, and during breath-holding, the involuntary subtle breathing movements can cause drifting in the measured waveform. As shown in Figure 9a, the heartbeat waveform exhibits a slow downward trend over time, attributed to the slight inhalation movement of the abdomen. The trend line of signal drift is in the low-frequency domain relative to the heartbeat curve, and high-pass filtering is an effective method to eliminate low-frequency interference. The waveform filtered by a high-pass filter with a cutoff frequency of 0.5 Hz is shown in Figure 9b. It can be observed that the drift phenomenon is eliminated, and the details of the heartbeat waveform are preserved.

Since the pumping of blood in the heart is mainly done by the ventricles, the prominent periodicity displayed in the waveform of the heartbeat reflects the periodicity of ventricular motion. The heart is mainly composed of ventricles and atria, and the motion of both is not a simple harmonic motion with a single frequency. The motion of the ventricles and atria combines to form the overall cardiac motion, resulting in small fluctuations in the periodic waveform. From the perspective of the frequency domain, the significant periodicity in the heartbeat waveform represents the fundamental frequency component of cardiac motion with the largest amplitude. The small fluctuations within each cycle represent the high-frequency components of the motion, which are part of the cardiac motion waveform and can also provide valuable information about the heart’s health status. However, the amplitude of the high-frequency components is relatively small and may be overshadowed by the fundamental frequency. By applying frequency domain filtering to remove the fundamental frequency of the heartbeat signal, the high-frequency components within the heartbeat cycle can be highlighted, allowing for a clearer examination of the details in the waveform.

The characteristic points of a signal are generally the extreme points of the waveform, representing the state of the signal at which it is about to change. In [22], cardiac activity was measured using a single-tone continuous wave radar, and the velocity waveform was obtained by differentiating the heartbeat waveform. A comparison was made between the velocity waveform and the ECG signal, noting that three extremal points in the velocity waveform corresponded to specific positions in the ECG waveform. The minimum velocity value (maximum in this paper) occurred within the QRS complex, while another velocity peak (minimum in this paper) corresponded to the end of the T wave. It was also observed that the first minimum preceding the minimum velocity always occurred at the beginning of the P wave. The experimental results in this paper reproduced the first two characteristic points, but the third one was an exception. The reason may be that differential calculation is very sensitive to small fluctuations. Noise and the degree of detail restoration of the waveform can lead to significant fluctuations in velocity waveforms, resulting in a lack of stability in the characteristic points of velocity waveforms.

**Figure 10:**
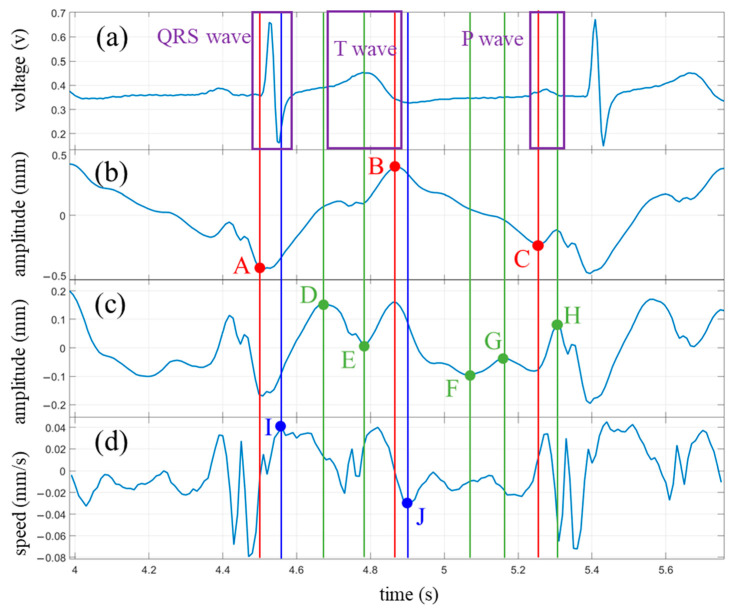
Experimental results under breath-hold condition and comparison with ECG waveforms. (**a**) ECG waveform; (**b**) Heartbeat waveform; (**c**) Heartbeat waveform after removing the fundamental wave; (**d**) Velocity waveform obtained by differentiating the heartbeat waveform. The annotations aligned with feature points identified on the heartbeat waveform are denoted in red, while those corresponding to points found on the waveform from which the fundamental wave have been removed are highlighted in green. Blue designates the locators tied to the feature points discovered on the velocity waveform.

To further explore cardiac health information within the radar measurement waveform, two complete heartbeat cycles (enclosed by the red box) were extracted from Figure 9b and magnified for display in Figure 10b. The synchronized ECG signal (Figure 10a), the waveform with the fundamental frequency removed (Figure 10c), and the velocity waveform (Figure 10d) are shown on the same time axis. Additionally, characteristic points of each waveform within a heartbeat cycle are marked.

The ECG waveform was annotated with a purple box to indicate the three main regions: the QRS complex, the T wave, and the P wave. The extremal points of the heartbeat waveform, along with their corresponding time coordinates, were marked with red dots and vertical lines, denoted as points A, B, and C. The extremal points of the waveform with the fundamental frequency removed were highlighted with green dots and vertical lines labeled as points D, E, F, G, and H. Among these, points E and H were shared between the two waveforms but exhibited more prominence in the waveform with the fundamental frequency removed. The characteristic points on the velocity curve, along with their respective time coordinates, were denoted with blue dots and vertical lines, representing points I and J.

In total, ten characteristic points were marked in Figure 10. However, some of these points, such as points F and G, may not appear in every measurement. During the measurement process, both the angle and distance between the radar and the subject can influence the waveforms, resulting in varying prominence levels of certain characteristic points.

The characteristic points marked in Figure 10 are analyzed below.

The characteristic point A occurs when the chest wall is nearest to the radar within a vibration cycle. At this moment, the ventricle reaches its maximum diastolic state. We can observe that point A falls within the QRS complex range of the ECG signal, roughly around the starting point of the R wave. Another point within the QRS complex is point I of the velocity waveform, which occurs slightly later than point A, close to the end of the QRS complex. This suggests that during the early stage of ventricular contraction, velocity reaches its maximum value. If we approximate the midpoint between points A and I as the central point of the R wave, we can calculate the RR interval between adjacent heartbeat cycles.

Point B is the opposite of point A, occurring when the chest wall is farthest from the radar, indicating the ventricle’s maximum contraction. This point is close to the end of the T wave in the ECG signal, slightly earlier than the T wave endpoint (point J) indicated in the velocity waveform. Within the time range of the T wave, we also have points D and E, with D approximating the starting moment of the T wave and E located within the central region of the T wave. Points D and J can be used to calculate the interval of the T wave.

Point C in the radar measurement waveform coincides with the onset of the P wave in the ECG signal, representing the late stage of ventricular diastole. From the vibration waveform, we can observe that before point C, the skin on the body surface moves in the opposite direction of the radar due to the influence of ventricular diastole. At point C, the direction of vibration starts to change and, after a brief duration of approximately 0.05 s, returns to its original direction. This change in vibration direction at point C may indicate a transient effect of atrial contraction that momentarily exceeds the influence of ventricular diastole on the body surface.

Point H occurs close to the endpoint of the P wave and can be used in conjunction with point C to estimate the approximate interval of the P wave. The central points of the P wave and the R wave can also be used to calculate the PR interval.

Points F and G fall within the TP interval of the ECG signal when the ventricle is in a diastolic state. Correspondingly, the ECG waveform is relatively flat during this period. Further exploration is required to unveil the significance of these two characteristic points.

By comparing with the ECG signal, it is evident that the radar-measured cardiac waveform provides information on the intervals of the P wave and T wave in the ECG signal, as well as the RR interval and PR interval. These intervals are closely related to the cardiac health. For example, changes in the RR interval, known as heart rate variability, can assess the balance between the sympathetic and parasympathetic nervous systems. The PR interval represents atrioventricular conduction time, and elongated intervals indicate conduction block, while shortened intervals may indicate pre-excitation syndrome.

#### 4.2.2. The Heartbeat Waveform under Normal Breathing State

**Figure 11:**
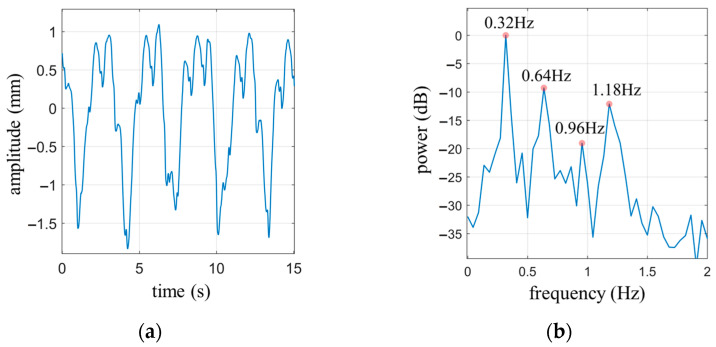
(**a**) Cardiopulmonary activity waveform under normal breathing state of the subject; (**b**) The power spectrum of (**a**).

The subject sits quietly in a chair, with the radar positioned 0.4 m away from the chest. The experiment was conducted under the normal breathing state of the subject. The measurement duration was set to 15 s. Figure 11a shows the measured cardiopulmonary activity waveform, and Figure 11b is the power spectrum of (a).

From Figure 11a, it can be observed that there are four complete respiratory cycles. Following the calculation rules for the waveform coverage area of the circumscribed rectangle mentioned earlier, the waveform coverage area for the four complete respiratory cycles is calculated as $S_{bre}=19.46$, while the area of the circumscribed rectangle is $S_{rec}=28.05$. The ratio between these two areas is r=0.694. Referring to Table 2, it can be determined that $N_{bre}=3$, indicating the presence of two harmonic components in the respiratory signal.

Figure 11b displays the power spectrum of the cardiopulmonary signal. It reveals that the peak frequency of the spectrum is 0.32 Hz, which corresponds to the fundamental frequency of respiration. The respective frequencies of the two respiratory harmonic components are 0.64 Hz and 0.96 Hz. If a high-pass filter with a cutoff frequency of 0.8 Hz is applied, two significant peaks can be observed in the passband at 0.96 Hz and 1.18 Hz. Based on the calculations of the respiratory signal harmonics, it is determined that 0.96 Hz represents the second harmonic frequency of the respiratory signal, while 1.18 Hz, as the most prominent frequency component among the remaining peaks, should correspond to the fundamental frequency of the heartbeat signal.

**Figure 12:**
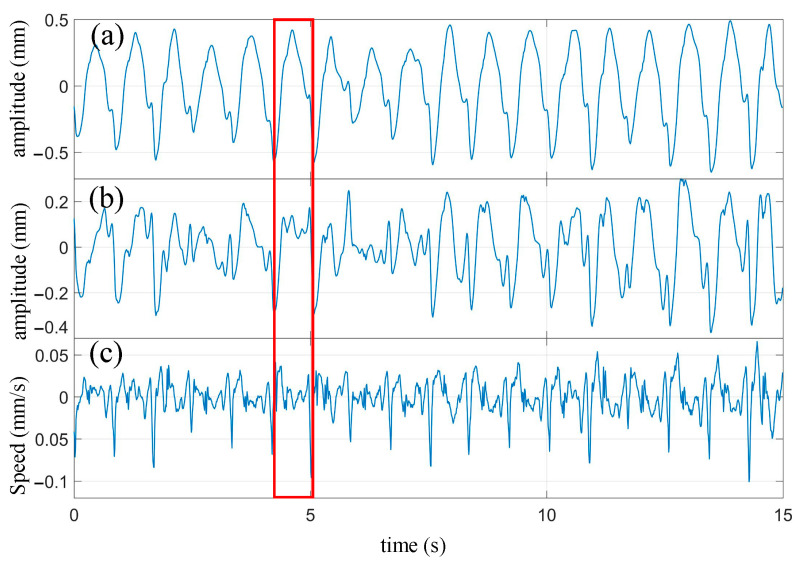
The processed waveform of heartbeat. (**a**) Heartbeat waveform extracted from cardiopulmonary activity waveform; (**b**) The waveform after removing the fundamental wave of heartbeat; (**c**) Velocity waveform obtained by differentiating the heartbeat waveform.

Applying the frequency domain filtering method with a cutoff frequency of 0.8 Hz, the measured signal was high-pass filtered. Simultaneously, the precise elimination of respiratory harmonics within the passband was achieved, resulting in the extraction of the heartbeat signal. Subsequently, the obtained heartbeat signal was processed by first applying the frequency domain filtering method to remove the fundamental component of the heartbeat signal. Then, the signal was differentiated to obtain the velocity waveform. The processed waveform is shown in Figure 12.

Compared with the heartbeat waveform measured during breath-holding in Figure 9b, it can be observed that the extracted heartbeat waveform from the cardiopulmonary signal measured under normal breathing conditions effectively captures the periodicity of the heartbeat. However, there are some minor details missing in the waveform. For instance, in Figure 9b, there is a notch near the peak of the rising portion of the waveform, which is mostly absent in the heartbeat waveform shown in Figure 12a. It should be noted that in the heartbeat waveform of Figure 12b, after removing the fundamental component, most of these notches are partially restored. Overall, the extracted heartbeat waveform from the cardiopulmonary signal measured under normal breathing conditions exhibits a significant reduction or even disappearance in the magnitude of minor fluctuations that represent waveform details. From a frequency domain perspective, these small fluctuations, compared to the fundamental frequency of the heartbeat, belong to the high-frequency domain. The respiratory motion leads to the loss or deformation of the high-frequency components of the extracted heartbeat signal.

**Figure 13:**
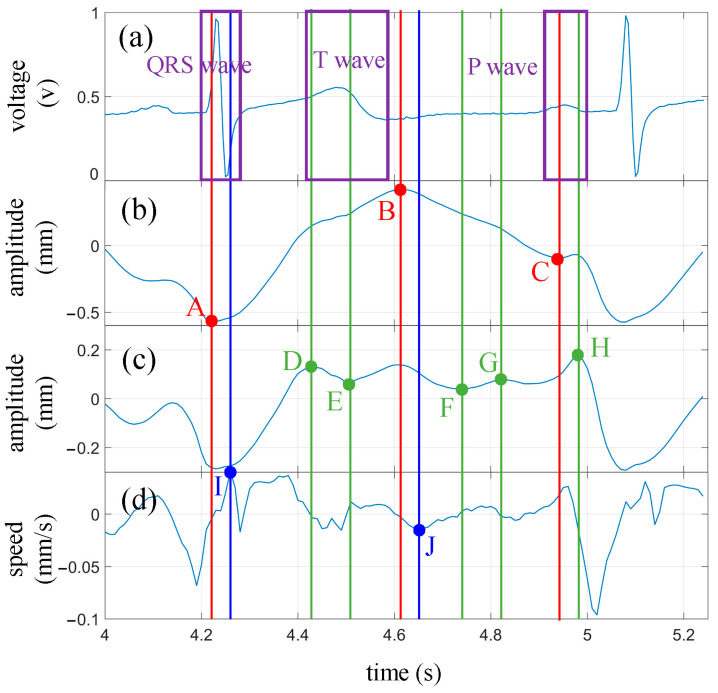
The processed waveform of heartbeat under normal breathing condition and comparison with ECG waveforms. (**a**) ECG waveform; (**b**) Heartbeat waveform; (**c**) Heartbeat waveform after removing the fundamental wave; (**d**) Velocity waveform obtained by differentiating the heartbeat waveform. The annotations aligned with feature points identified on the heartbeat waveform are denoted in red, while those corresponding to points found on the waveform from which the fundamental wave have been removed are highlighted in green. Blue designates the locators tied to the feature points discovered on the velocity waveform.

One heartbeat cycle was extracted from Figure 12 (enclosed by the red box) and compared with the synchronized ECG waveform, as shown in Figure 13. It can be seen that similar characteristic points as those obtained during breath-holding can be identified. The corresponding times for each characteristic point are as follows: A: 4.23 s; B: 4.61 s; C: 4.94 s; D: 4.13 s; E: 4.51 s; F: 4.74 s; G: 4.82 s; H: 4.98 s; I: 4.26 s; J: 4.65 s. The P wave interval can be computed as 0.11 s, the T wave interval as 0.52 s, and the PR interval as 0.13 s, based on these extreme points. Additionally, in combination with the extreme points of the next cycle, an RR interval of 0.83 s can be derived. It is worth noting that some of these extreme points are not prominently pronounced, such as points F and G. The amplitude of the fluctuations at these characteristic points is weak during breath-holding and even more unstable under normal breathing conditions. These experiments demonstrate that meaningful characteristic points can still be clearly identified from the extracted heartbeat waveform, even under normal breathing conditions.

### 4.3. Comparison of Performance with Other Works

Most radar-based cardiac motion studies focus on the periodicity of the heartbeat, such as heart rate or heart rate variability [26,27,28,45,46,47], and lack an analysis of the movement characteristics within a heartbeat cycle as shown in Table 3. Some studies have utilized the first-order and second-order derivatives of the heartbeat waveform to extract the velocity and acceleration waveforms, which are then compared to the synchronously measured ECG signal to identify matching features [22,48,49]. Ref. [48] uses a 24 GHz continuous wave and identifies five feature points. Ref. [49], using 60 GHz continuous wave, obtained a more detailed heartbeat waveform, and eight feature points were identified. However, since noise may produce significant deviations in the results of differentiation operations, using velocity and acceleration waveforms to extract feature points has the drawback of poor stability. In this work, the high-frequency components with small amplitudes are highlighted by removing the fundamental wave of the heartbeat waveform, and ten feature points were identified, which can provide more details for extracting heart health information.

## 5. Discussion

**Figure 14:**
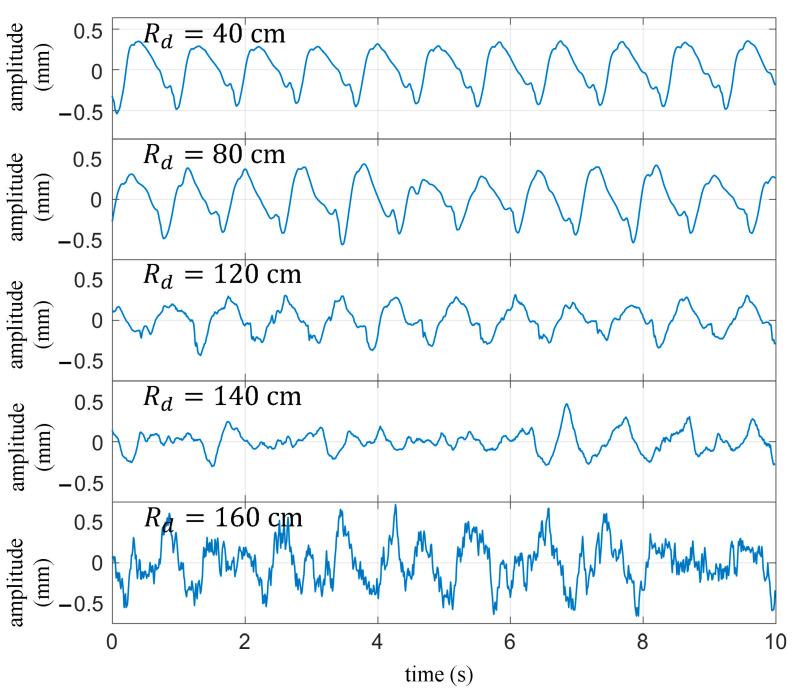
Under normal breathing conditions, the extracted heartbeat waveform varies with the distance between the target and the radar.

During practical measurements, the quality of the signal is influenced by various factors, such as the radar transmission power, the angle between the radar beam and the human body, and the distance between the radar and the chest. In real-world applications, $R_{d}$ is a variable quantity that can significantly impact the measurement results of the cardiopulmonary signal. Figure 14 illustrates the extracted heartbeat signals under normal breathing conditions with different values of $R_{d}$. The measurement process adopted a single variable method to minimize the influence of other factors, keeping the external environment constant, maintaining the same posture for the subjects, and keeping the radar transmission power and the angle relative to the human body unchanged. It can be observed that as Rd increases, the heartbeat waveform gradually distorts. When $R_{d}$ is equal to 120 cm, the details of the signal waveform experience severe distortion but still exhibit periodicity of the heartbeat. However, when $R_{d}$ exceeds 140 cm, the waveform can no longer reflect the periodicity of the heartbeat.

The beam divergence causes the received signal power to rapidly decrease with increasing distance, resulting in a reduction in signal-to-noise ratio. Beam focusing and enhancing transmission power are effective ways to improve the signal-to-noise ratio of received signals, which can increase the effective measurement distance.

## 6. Conclusions

This work applies a high-precision motion detection algorithm to measure cardiac motion. To address the issue of interference from respiratory signal harmonics in extracting the heartbeat signal, an algorithm is proposed that utilizes the ratio of the respiratory signal waveform coverage area to the circumscribed rectangle area to determine the harmonic components. By removing the respiratory harmonic components within the frequency range of the heartbeat signal, accurate extraction of the heartbeat waveform is achieved. Additionally, to overcome the limited information extraction challenge in radar-measured heartbeat waveforms, frequency domain filtering is employed to remove the fundamental component of the heartbeat signal. The resulting waveform is then compared with the heartbeat waveform, heartbeat velocity waveform, and synchronized ECG signal. The corresponding relationship between certain extreme points of the waveform and the characteristic positions of the ECG signal is analyzed, obtaining information related to cardiac health.

The noncontact, fast, and unobtrusive monitoring advantages of radar make it particularly suitable for daily health monitoring. By exploring the different characteristics of individuals with cardiac diseases and healthy individuals in radar-based cardiopulmonary signals, the diagnosis of cardiac diseases can be achieved. This can be applied to home or nursing home health monitoring, aiming to enable early detection of cardiac diseases.

## Figures and Tables

**Table 1:** Parameters related to respiration and heartbeat.

Item	Fundamental Frequency Range (Hz)	Frequency per Minute (Hz/min)	Amplitude of Chest Wall (mm)
Respiration	0.13~0.4	7.8~24	4~12
Heartbeat	0.83~3.3	49.8~198	<0.6

**Table 2:** The correspondence between $N_{bre}$ and the r.

$N_{bre}$	1	2	3	4	5	6	7	8	9	10
r	0.5	0.625	0.688	0.727	0.754	0.774	0.791	0.804	0.815	0.824

**Table 3:** Comparison with recent works.

Ref.	Waveform	Single-Channel	ISM Band	The Detection Content of Cardiac Activity	Number of Feature Points	Type of Waveform Used
[26]	CW	NO	NO	Periodicity	None	HW ^1^
[27]	CW	NO	NO	Periodicity	None	HW
[28]	SFCW	NO	NO	Periodicity	None	HW
[45]	FMCW	YES	NO	Periodicity	None	HW
[46]	FMCW	NO	NO	Periodicity	None	HW
[47]	FMCW	NO	NO	Periodicity	None	HW
[22]	CW	NO	YES	Periodicity and Feature Points	3	HW and SW ^2^ and AW ^3^
[48]	CW	NO	YES	Periodicity and Feature Points	5	HW and SW and AW
[49]	CW	NO	YES	Periodicity and Feature Points	8	HW and SW and AW
This work	FMCW	YES	YES	Periodicity and Feature Points	10	HW and SW and HWRFW ^4^

^1^ HW = Heartbeat waveform. ^2^ SW = Speed waveform. ^3^ AW = Acceleration waveform. ^4^ HWRFW = Heartbeat waveform after removing the fundamental wave.

## Data Availability

Data are contained within the article.
